# Individual-level deviations from normative brain morphology in violence, psychosis, and psychopathy

**DOI:** 10.1038/s41398-025-03343-1

**Published:** 2025-04-02

**Authors:** Unn K. Haukvik, Thomas Wolfers, Natalia Tesli, Christina Bell, Gabriela Hjell, Thomas Fischer-Vieler, Nina Bang, Ingrid Melle, Ole A. Andreassen, Kirsten Rasmussen, Ingrid Agartz, Lars T. Westlye, Christine Friestad, Jaroslav Rokicki

**Affiliations:** 1 Adult Psychiatry Department, Division of Mental Health and Addiction, Institute of Clinical Medicine, University of Oslo, Oslo, Norway; 2 Centre for Research and Education in Forensic Psychiatry (SIFER), Oslo University Hospital, Oslo, Norway; 3 Department of Psychiatry and Psychotherapy, University of Tübingen, Tübingen, Germany; 4 German Center for Mental Health, Tübingen, Germany; 5 Norwegian Centre for Mental Disorders Research (NORMENT), Division of Mental Health and Addiction, Oslo University Hospital, and Institute of Clinical Medicine, University of Oslo, Oslo, Norway; 6 Department of Psychiatry, Oslo University Hospital, Oslo, Norway; 7 Department of Psychiatry, Østfold Hospital Trust, Graalum, Norway; 8 Vestre Viken Hospital Trust, Division of Mental health and Addiction, Drammen, Norway; 9 St.Olavs Hospital, Centre for research and education in forensic psychiatry, Trondheim, Norway; 10 Norwegian University of Science and Technology (NTNU), Department of Mental Health, Trondheim, Norway; 11 Section for Clinical Psychosis Research, Division of Mental Health and Addiction, Oslo University Hospital, Oslo, Norway; 12 Centre for Precision Psychiatry, Division of Mental Health and Addiction, Oslo University Hospital, and University of Oslo, Oslo, Norway; 13 Division of Mental Health and Substance Abuse, Diakonhjemmet Hospital, Oslo, Norway; 14 Centre for Psychiatry Research, Department of Clinical Neuroscience, Karolinska Institutet & Stockholm Health Care Services, Stockholm Region, Stockholm, Sweden; 15 Division of Mental Health and Addiction, Institute of Clinical Medicine, University of Oslo, Oslo, Norway; 16 Department of Psychology, University of Oslo, Oslo, Norway; 17 University College of Norwegian Correctional Service, Lillestrøm, Norway

**Keywords:** Human behaviour, Diagnostic markers, Schizophrenia

## Abstract

Neuroimaging research has shown brain morphological abnormalities associated with violence and psychosis, but individual differences are substantial and results not consistent across studies. Normative modeling of brain MRI-features facilitates a systematic mapping of individual brain characteristics of complex phenotypes also in small samples but has not yet been applied to forensic psychiatry populations. We explored brain heterogeneity in persons with a history of severe violence with a comorbid schizophrenia spectrum disorder (SSD-V; *n* = 38), non-violent persons with schizophrenia spectrum disorders (SSD-NV; *n* = 138), persons with a history of severe violence without comorbid schizophrenia spectrum disorder (nonSSD-V; *n* = 20), and healthy non-violent participants (HC; *n* = 196) from lifetime normative trajectories of cortical thickness, surface area, and subcortical volumes. Normative models based on Freesurfer derived regions of interest from 58,836 individuals were used to investigate individual deviances, group differences, and associations to psychopathy traits. We found overall heterogeneous patterns of individual deviations from the norm, which were most prominent for regions within the collateral transverse sulcus, lingual gyrus, and cerebellum among SSD-V, a pattern that differed from SSD-NV (parieto-occipital and suborbital sulci), and nonSSD-V (paracentral and middle frontal regions). We found no significant associations to psychopathy traits. By applying normative modeling, we demonstrate heterogeneous patterns of brain morphometry deviations associated with violence and psychosis. While the results warrant replication, studies addressing individual brain deviations may contribute to improved understanding of the neurobiological underpinnings of comorbid violence and psychosis, which ultimately may have clinical impact on treatment and forensic psychiatric evaluations.

## Introduction

Violence committed by persons with severe mental disorders is a tragedy for the people involved, the treating health services, and the society at large. A key to prevent such events is to understand the complex biopsychosocial underpinnings of violence associated with severe mental disorders and their interaction with each other [1]. Over the last decades, neuroimaging research has tried to map the anatomy and functional characteristics associated with violence committed by persons with severe mental disorders (see [2] for review). Magnetic resonance imaging (MRI) studies have shown volumetric reductions of the orbitofrontal [3] and anterior cingulate [4] cortex, the amygdala and hippocampus [5], including their subfields and nuclei [6, 7], as well as cortical folding and thickness abnormalities [8–10] in persons with psychosis and a history of severe violence. This pattern of brain deviations aligns with regions and circuits involved in fear [11], reward [12], empathy [13], the processing of visual stimuli [14], and the formation of positive psychosis symptoms including hallucinations [15] and delusions [16]. The findings vary across studies, which is expected considering the diversity of the violence and psychosis phenotypes [17], but the traditional neuroimaging research statistical methodology comparing group averages does not account for this heterogeneity. As such, putative individual brain deviations and patterns pointing toward distinct phenotypic neurobiological underpinnings might remain undiscovered.

Following recent advances in computational neuroscience, neuroimaging research has moved towards analyzing datasets from large publicly available databases or collaborative mega-analyses of data from individual research centers worldwide, which facilitates the disentanglement of neurobiological underpinnings of psychopathology on a large scale [18]. However, while most of the current research is conducted on the group level, clinicians and forensic experts must make decisions on the individual level (*n* = 1). Moreover, neuroimaging studies show great heterogeneity and associations to psychopathology are subtle [19]. To supplement conventional group-based analyses, statistical methods have been developed to model deviations from the norm to address this heterogeneity, and the subtle brain abnormalities that have been associated with mental health disorders, on the individual level. The core concept of these models is based on pediatric growth charts, where individual variation is quantified against the growth information from a large reference population [20]. This approach has been translated into mapping individual brain characteristics to reference maps of age-related brain anatomy [21], which provides an alternative to the classical case-control or cluster analyses in neuroimaging research [22]. By using normative models, heterogeneous patterns of brain deviations have been mapped in schizophrenia and bipolar disorder [23], in antipsychotic naïve first episode psychosis patients [24], and associated with negative symptoms longitudinally [19]. Moreover, aggression has been linked to age-related brain deviations specifically in the amygdala in youth with disruptive behavior disorders [25] and trait impulsivity and reward-related brain responses, to individual brain deviations associated with clinical symptoms [26]. As such, using normative modeling in forensic psychiatry neuroimaging studies may overcome inherent challenges related to the small subject samples (due to concerns related to severe psychopathology, ability to consent, and security measures) and serve as a powerful tool not only to investigate underlying mechanisms of violent behavior in psychosis, but also to provide clinicians with individual level neurobiological information that together with other clinical information/characteristics may inform treatment selection and targeted interventions at the individual level.

The objective of this study is to assess the potential of normative modeling in forensic psychiatry neuroimaging research by investigating individual patterns of deviations from the norm in persons with a history of severe violence and schizophrenia spectrum disorder (SSD-V), in persons with a schizophrenia spectrum disorder but no history of violence (SSD-NV), persons with a history of severe violence without schizophrenia spectrum disorder (nonSSD-V), and healthy controls with no history of violence (HC). We hypothesize that there will be high inter-individual anatomical variability in persons with SSD-V, but with more extreme deviations in regions previously associated with violence in severe mental disorders such as the amygdala, hippocampus, orbito-frontal cortex, and anterior cingulate cortex ([2] for review). Moreover, we expect the pattern of deviations in SSD-V to differ from SSD-NV, where we expect more extreme deviations in regions previously associated with schizophrenia, such as the prefrontal cortices, hippocampus, and the basal ganglia [23, 27, 28]. Finally, we hypothesize that psychopathy traits will be associated with specific patterns of extreme deviations in both SSD-V and nonSSD-V [29].

## Methods

### Participants

Participants (*n* = 782 male/608 female) were recruited from the Oslo area as part of four studies: the Thematically Organized Psychosis study (TOP) [30], the STROKEMRI study [31], The Youth-TOP study (uTOP) [32], and The Forensic Psychiatry study (sTOP) [33]. 998 healthy controls (390 male/608 female) were used as a calibration set to adjust the model to our data [20]. The remaining participants consisted of four groups: (1) persons with a history of severe violence and comorbid schizophrenia spectrum disorder (SSD-V, *n* = 38; i.e. schizophrenia *n* = 35, schizoaffective disorder *n* = 1, schizophreniform disorder *n* = 1, other psychosis *n* = 1), and three control groups: (2) persons with a history of severe violence and no comorbid psychosis disorder (nonSSD-V, *n* = 20), (3) persons with no history of violence but with a schizophrenia spectrum disorder (SSD-NV *n* = 138; i.e. schizophrenia *n* = 87, schizoaffective disorder *n* = 17, schizophreniform disorder *n* = 7, other psychosis *n* = 27), and (4) healthy controls (HC, *n* = 196) matched by age and scanner. All individuals were male due to the scarcity of eligible women within the participating forensic psychiatry units and prisons. The participants had no history of head trauma that resulted in loss of consciousness, and no present or recent somatic disease that might have impacted brain morphology.

The inclusion criteria for *persons with a history of violence (SSD-V and nonSSD-V)* were age between 18 and 70 years and a court or hospital record verified episode of severe violence, defined as homicide, attempted homicide, or physical (including sexual) violence towards other persons. The SSD-V group had in addition a DSM-IV diagnosis of schizophrenia spectrum disorder and were sentenced or committed to compulsory mental health care in high security hospital wards. The nonSSD-V group had no comorbid psychosis disorder, and all served a preventive detention sentence in a high security prison. The *schizophrenia spectrum disorder without a history of violence* (SSD-NV) inclusion criteria required participants to be between 12 and 65 years old, with a DSM-IV diagnosis of schizophrenia spectrum disorder and no history of severe violence. The participants were included from hospital wards and out-patient clinics. *Healthy participants* were either drawn from the Norwegian national population registry (TOP, uTOP), or recruited by newspaper ads and word of mouth (STROKEMRI). The inclusion criteria were age 12–18 (uTOP), 18–65 (TOP), or 20–88 (STROKEMRI) and no history of neurological or severe mental disorders.

The Norwegian Regional Committee for Medical and Health Research Ethics approved the project which was conducted in accordance with the Helsinki declaration. All participants submitted written informed consent.

### Clinical assessment

Clinical characterization was performed by specially trained psychiatrists, medical doctors, and psychologists. Structured diagnostic assessment was performed with the SCID-I [34] (18 years or above), and the KIDDIE-SADS present and lifetime version [35] (below 18 years). The positive and negative symptom scale (PANSS) [36] was used to assess psychosis symptoms, the Wechsler Abbreviated Scale of Intelligence (WASI) for IQ [37], and substance use with AUDIT [38] and DUDIT [39]. Psychopathy traits were assessed with the Psychopathy Checklist Revised (PCL-R) supplemented by hospital records and court documents [40]. The HC were assessed for mental disorders using the Primary Care Evaluation of Mental Disorders [41]. Use of antipsychotic medication was calculated based on daily defined doses according to the World Health Organization (https://www.whocc/atc_ddd_index/).

### MRI data acquisition

T1-weighted volumes were collected on two GE 3 T scanners located at the Oslo University hospital, Norway: (1) GE Signa HDxt scanner with a standard 8-channel head coil, using a sagittal 3D fast spoiled gradient echo (FSPGR) sequence with the following parameters: repetition time (TR) = 7.8 ms, echo time (TE) = 2.9 ms, flip angle 12°, slice thickness 1.2 mm, 166 sagittal slices, field of view (FOV) 256 × 256 mm, acquisition matrix 256 × 192 mm, voxel size = 1 × 1 × 1.2 mm^3^ and (2) DiscoveryTM-MR750 scanner with the vendor’s 32-channel head coil, using an inversion recovery-fast spoiled gradient echo sequence (BRAVO) with the following parameters: TR = 8.16 ms, TE = 3.18 ms, TI = 450 ms, flip angle = 12°, FOV = 256 mm, acquisition matrix = 256 × 256 mm, 188 sagittal slices, slice thickness = 1.0 mm, voxel size = 1 × 1× 1 mm^3^. MRI data was available from 837 male participants and 628 female participants. Among these, 52 males and 20 females were excluded due to insufficient T1w image quality, and 3 additional male individuals were excluded due to spurious abnormal brain findings.

### Quality control and preprocessing

Image quality checking was carried out in two steps. First, all T1w images were processed using MRIqc [42]. Then two trained raters visually examined the scans marked for exclusion by MRIqc pipeline (NT and JR). The quality of the area and thickness of cortical maps was assessed in a comprehensive visual evaluation of lateral and medial snapshots of all maps. If the surface values had negative values, unusual patterns, or a significant value imbalance across hemispheres, the individual was removed. Freesurfer v7.1 was used to process data that passed quality control. Cortical thickness and area was summarized using the Destrieux atlas parcellation scheme [43]. Subcortical volume segmentation was based on a probabilistic atlas with priors for 31 brain structures [44, 45]. The ROIs used to train the initial normative models determined the selection of parcellation atlases for cortical and subcortical regions.

### Normative models

We used pretrained normative models from https://github.com/predictive-clinical-neuroscience/braincharts. Briefly, models were trained using data of 58835 individuals aged 2–100 from 82 sites. To model non-linear effects accurately, normative models were trained using Bayesian linear regression with likelihood warping using sinarcsinsh function [46]. The dependent variable was set to the ROI values of volume, area, or thickness, while the independent variables were age and scanner. To summarize the cortical area and thickness, we used the regions described in a high-resolution Destrieux atlas [43]. Models were both trained and applied to our data using python 3.8 and PCNtoolkit package (version 0.20). Further details on normative models are described in [20]. To account for the absence of scanner data used to acquire data in the model, an additional harmonization step was implemented. An unmatched group of healthy controls (*n* = 998) was utilized to fine-tune model parameters, enabling the application of normative models to unseen sites.

### Statistical analysis

To summarize inter-individual variation within each group (SSD-V, SSD-NV, nonSSD-V and HC), we computed deviation scores (Z-scores) by separating them into positive and negative deviations. The percentage of subjects within each group with extreme positive (Z > 2) and negative (Z < −2) deviations at a given ROI were compared and reported [20]. Further, we examined which ROIs consistently exhibited extreme deviations in each modality (cortical area and thickness, subcortical volume) by quantifying the percentage of extreme deviations observed in each ROI for each diagnostic group. Then, we quantified the number of extreme deviations per individual to compare if participants from all diagnostic groups had a higher count of extreme deviations in general as compared to healthy controls.

Subsequently, for completeness, we conducted case-control group difference testing on the deviation scores. The R package matchIt was used to match age and scanning sites between HC and diagnostic groups using logistic regression distance with a (1:1) matching ratio [47]. General linear models (GLM), as implemented in the permutation analysis of linear models (PALM) toolkit [48], were used to perform the between-group statistical analyses, controlling for the effects of age and demeaning both data and independent variables in the design matrix. Additionally, for analyses contrasting volumes and areas, we included intracranial volume (ICV). We used family wise error correction to account for multiple corrections: the number of ROIs, three modalities (cortical area and thickness, and subcortical volume) and 8 contrasts [49]. Additionally, we computed Cohen’s d effect sizes.

To determine whether there were group differences in the frequency of extreme positive and negative deviations, we performed permutations. The input data consisted of the number of extreme positive and negative deviations per individual, aggregated across all modalities. Specifically, we tested if the diagnostic groups had more negative deviations as compared to healthy controls and each-other, and if the opposite pattern was held for extreme positive deviations.

Finally, to examine the association between PCL-R scores and deviation scores in individuals with a history of violence, we used the GLM in the PALM toolbox with 10,000 permutations. The deviation scores of each ROI were treated as the dependent variable, while the PCL-R total score, age, psychosis status, and ICV (for area and volume) were included as independent variables.

## Results

### Demographics

Demographic characteristics are summarized in Table 1. Groups differed significantly on age, IQ score, and substance use (DUDIT), but not in alcohol use (AUDIT) and PANSS scores.

**Table 1:** Demographic and clinical characteristics.

	SSD-NV (*n* = 138)	SSD-V (*n* = 38)	nonSSD-V (*n* = 20)	HC (*n* = 196)	Total (*n* = 392)	*P*-value *F-test*
Age (years)						
Mean (SD, min-max)	29.0 (8.7, 15.1–57.8)	34.7 (8.9, 19.2–54.1)	42.4 (14.4, 9.2- 71.0^a^)	31.9 (10.0, 15.7–70.9)	31.7 (10.2, 15.1–71.0)	*p* < 0.001 *F* = 13
Wechsler abbreviated scale of intelligence (WASI-IQ)						
Mean (SD) n128/19/17/152	102 (14.4)	93.8 (15.1)	102 (12.8)	114 (11.0)	107 (14.4)	*p* < 0.001 *F* = 28
Positive and negative syndrome scale total (PANSS)						
Mean (SD) *n* = 137/34/20/0	60.8 (17.1)	64.6 (18.9)	39.4 (11.2)	N/A	59.3 (18.2)	*p* < 0.001 *F* = 16
Psychopathy Checklist Revised (PCL-R)						
Mean (SD) *n* = 0/34/x/0	N/A	18.9 (8.15)	20.8 (7.84)	N/A	19.9 (7.92)	*p* = *0.505* *t* = *−0.67*
Use of antipsychotic medication (DDD)						
Mean (SD) *n* = 123/34/0/0	1.06 (0.834)	1.58 (0.790)	N/A	N/A	1.17 (0.849)	*P* = *0.002* *t* = *−3.2*
Drug use disorders identification test (DUDIT)						
Mean (SD) *n* = 124/30/0/129	5.56 (8.88)	9.47 (10.1)	N/A	0.51 (1.63)	3.67 (7.47)	*p* < 0.001 *F* = 30
Alcohol use disorders identification test (AUDIT)						
Mean (SD) *n* = 122/31/0/129	6.60 (6.35)	4.81 (4.6)	N/A	6.05 (3.51)	6.15 (5.05)	*p* = 0.2 *F* = 1.6

*SSD-NV* schizophrenia spectrum disorder patients without history of violence, *SSD-V* schizophrenia spectrum disorder patients with history of violence, *nonSSD-V* participants with history of violence and without schizophrenia spectrum disorder, *HC* healthy controls, *SD* standard deviation.

^a^The person was 70 years and 11 months at scanning time.

### Extreme deviations from the normal trajectory

**Fig. 1:**
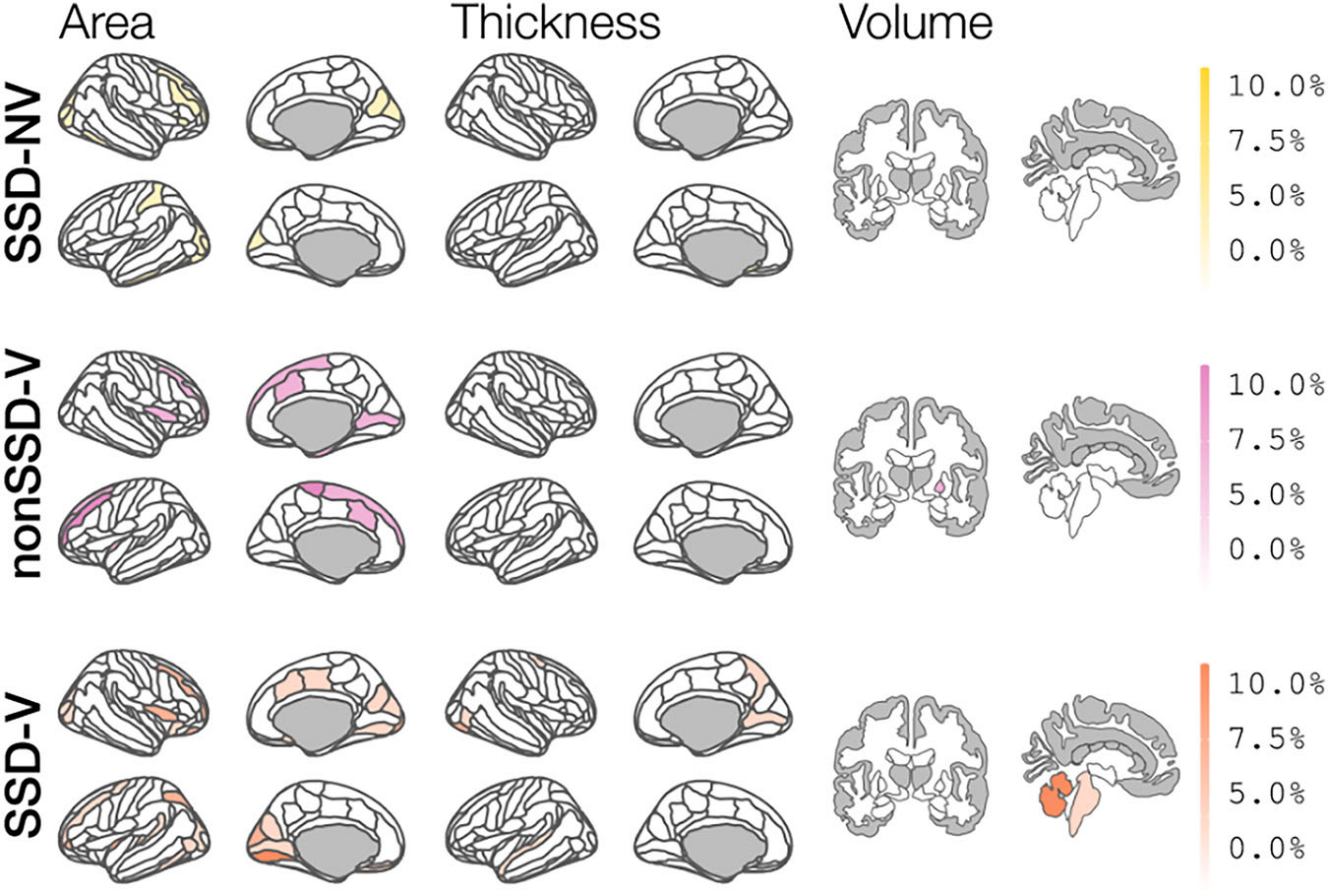
Relative frequency of individuals exhibiting extreme negative deviations (Z < −2) in specific ROIs. Regions with a minimum deviation frequency of 5, 7.5, and 10% are distinguished by progressively darker shades. Deviations are consistently observed in frontal lobe ROIs across all groups, whereas the SSD group also displays deviations in the occipital lobe. Notably, these patterns correspond to less than 5% of the individuals in the total sample. SSD-NV schizophrenia spectrum disorder patients without history of violence, SSD-V schizophrenia spectrum disorder patients with a history of violence, nonSSD-V participants with history of violence and no schizophrenia spectrum disorder, HC healthy participants.

Figure. 1 illustrates the differences in deviations patterns from the typical age-related trajectory among study groups, detailing cortical area, thickness, and subcortical volumes.

On average, SSD-V displayed extreme negative deviations in 2.6% of the cortical area regions. In contrast, individuals with SSD-NV and nonSSD-V exhibited, on average, a 3.1% rate of extreme negative deviations within cortical area regions (Supplementary Table S1), while HC showed a 1.5% deviation rate. Extreme negative cortical thickness deviations ranged from 0.93% (nonSSD-V) to 1.33% (SSD-NV). Additionally, persons with a history of violence, whether with SSD-V or nonSSD-V, showed more pronounced negative deviations in subcortical regions, amounting to 2.1 and 3.0%, respectively, indicating three-to-five-fold increase as compared to HC, who had a rate of only 0.6%. When examining extreme positive deviations, HC presented rates of 1.5% in cortical area, 1.6% in thickness, and 1.2% in subcortical volume regions. SSD-V, SSD-NV, and nonSSD-V generally had fewer positive deviations, except for the SSD-V group’s rate of 2.1% in subcortical volumes (Supplementary Table S1). These were significant between SSD-V and HC (*p* = 0.0190, *d* = 0.58) regarding the frequency of positive deviations, and between SSD-NV and HC (*p* = 0.0086, *d* = 0.31) for negative deviation frequencies, (Supplementary Table S2). Compared to HC, all diagnostic groups comprised a higher proportion of individuals with extreme deviations across all measurements (Supplementary Fig. S1).

### Regional patterns of brain deviations

Figure. 1 illustrates the regional patterns of extreme negative deviations across diagnostic categories. For patterns of extreme positive deviations see Supplementary Fig. S1.

**Fig. 2:**
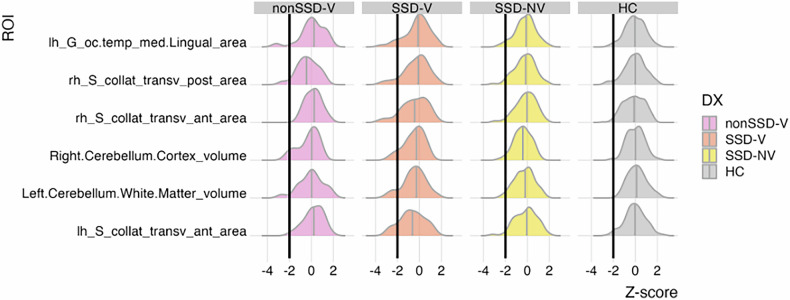
Z-scores indicating deviations from the normative curve for regions with at least 10% of extreme negative deviations in the SSD-V group. The threshold denoting extremely negative deviations is represented by a black vertical line (Z < −2). SSD-NV schizophrenia spectrum disorder patients without history of violence, SSD-V schizophrenia spectrum disorder patients with a history of violence, nonSSD-V participants with history of violence and no schizophrenia spectrum disorder, HC healthy participants.

In summary, we found that 89.5% of SSD-V and 65% of nonSSD-V had at least one extreme deviation from the normative range, but the percentage of extreme deviations for any given region did not exceed 13.2% for SSD-V and 20% for nonSSD-V (Supplementary Tables S3, S4, for negative and positive deviations respectively). While all groups exhibit deviations in the prefrontal cortex, both SSD-V and SSD-NV additionally demonstrate notable negative deviations in regions of the occipital lobe. Within SSD-V, the regions exhibiting the highest proportion of subjects with extreme negative deviations (Fig. 2) include: the left anterior (13.2%) and the right posterior (10.5%) segments of the collateral transverse sulcus area, the medial area of the lingual gyrus in the left occipito-temporal region (10.5%), and the volume of the right cerebellar cortex (10.5%). The 15 regions with the highest proportion of negative deviations are listed in Supplementary Table S5. Conversely, in the SSD-NV group, the regions that were most associated with extreme negative deviations were found in the right hemisphere’s parieto-occipital and suborbital sulcus areas, each affecting 7.2% of individuals (Supplementary Table S6). Among the nonSSD-V, the highest percentages of extreme negative deviations were noted in the left hemisphere’s paracentral lobule and sulcus, and the middle frontal gyrus, impacting 20 and 15% of subjects, respectively (Supplementary Table S5).

**Fig. 3:**
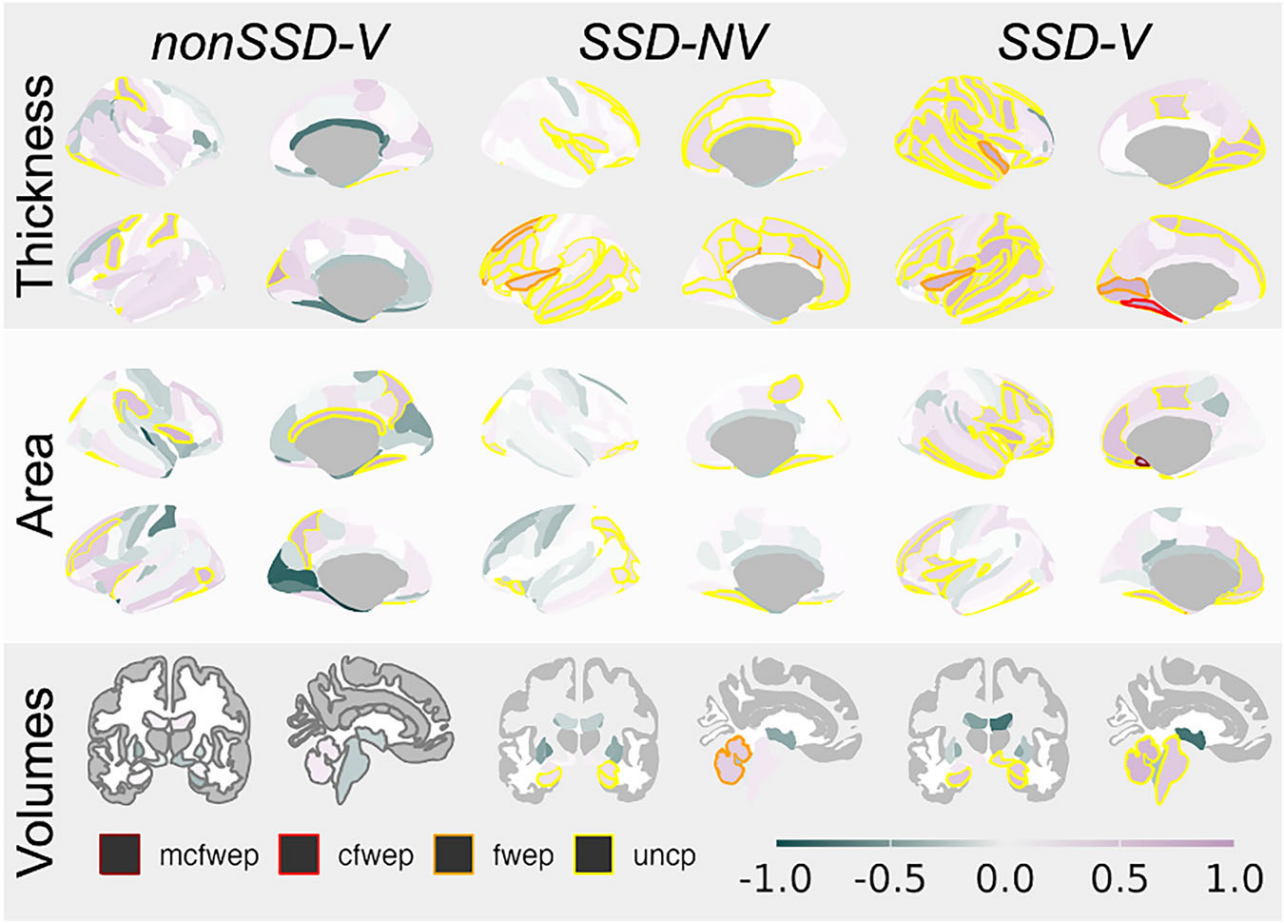
Pairwise difference in Z-scores between diagnostic groups and age matched healthy controls with ratio (1:1). ROIs with significant results are marked with contour lines: *yellow (uncp)* uncorrected, *orange (fwep)* FDR corrected for the number of ROIs, *red (cfwep)* FDR corrected for the number of ROIs and number of contrasts and *crimson (mcwep) p*-value FDR corrected for number of ROIs, contrast and modalities. Shades presents Cohen’s *d* of group differences on the deviation scores.

Additionally, we extended our analysis to include not only extreme deviations but also more subtle variations, by contrasting each diagnostic group’s deviation scores with HC (Fig. 3 and Supplementary Table S7). At the strictest correction level, SSD-V had significantly higher mean z-score values than HC for negative deviations in the right subcallosal area (*p*_*mc-fwe*_ = 0.020, *t* = 4.5, *d* = 1.17) and when controlling for the number of ROIs and contrasts (but not imaging modalities) also for the left medial occipito-temporal and lingual sulcus thickness (*p*_*c-fwe*_ = 0.022, *t* = 4.3, *d* = 0.99). These regions also differed between SSD-V and nonSSD-V when corrected only for the number of ROIs (but not contrasts and imaging modalities).

Moreover, SSD-NV exhibited significantly larger positive deviations in the volume of the left inferior lateral ventricle (*p*_*c-fwe*_ = 0.0085, *t* = 4.2, *d* = 0.61) and in pallidum volume (*p*_*c-fwe*_ = 0.025, *t* = 4.0, *d* = 0.57) when compared to HC. Among the 332 regions tested across 8 contrasts (2656 tests in total) these regions remained significant after multiple comparison adjustment (Fig. 3, Supplementary Table S8). Differences between clinical groups were observed at the level of correction for number of ROIs only, with nonSSD-V showing significantly more deviations in the left superior medial and lingual thickness and anterior transversal collateral area compared to SSD-V, and SSD-V compared to SSD-NV in the choroid plexus. For completeness, the analyses were repeated with the whole healthy sample as a control group rather than the (1:1) matching (Supplementary Table S9, Supplementary Fig. S3).

### Associations with psychopathy traits

**Fig. 4:**
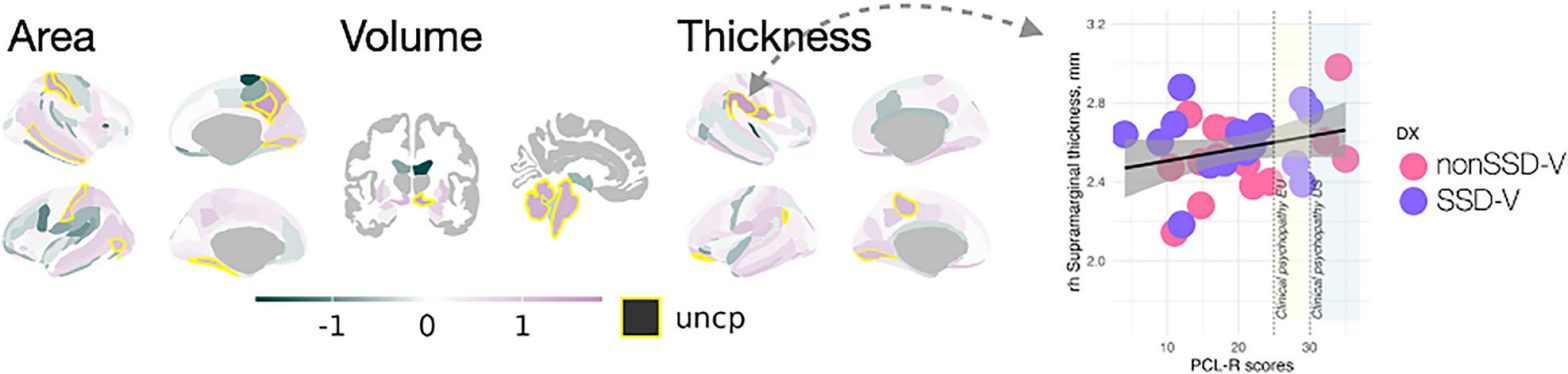
Associations between deviations from norm and psychopathy traits (PCL-R). ROIs fill color represents effect size (Cohen’s *d*) of association. While ROIs contour represents significance of the association: *yellow (uncp)* - uncorrected *p* values for the number of ROIs, contrast and modalities. None of the results remained significant after adjustment for multiple comparisons, but the overall, heterogeneous PCL-R association pattern is positive (in blue). Right most panel presents the association between psychopathy traits (PCL-R scores) and cortical thickness in the right hemisphere supramarginal gyrus, among persons with a history of violence with (purple) or without (pink) a schizophrenia spectrum disorder.

PCL-R scores did not significantly differ between SSD-V and nonSSD-V (*t* = 0.505, *p* = 0.45). We found no significant associations between psychopathy scores and brain morphology deviations, but an overall heterogeneous pattern with predominantly positive associations, which was trend level (uncorrected) significant in the right subparietal sulcus area (*d* = 1.8, *p*_*unc*_ = 0.0041) and parieto-inferior supramarginal gyral thickness (*d* = 1.8, *p*_*unc*_ = 0.0056) (Fig. 4). A full list of the associations for each modality is provided in the Supplementary Table S10.

## Discussion

In the present study, we used brain imaging and normative modeling to map individual brain morphological deviations in persons with a history of violence and comorbid schizophrenia spectrum disorder (SSD-V), non-violent persons with a schizophrenia spectrum disorder (SSD-NV), persons with a history of violence but no comorbid schizophrenia spectrum disorder (nonSSD-V), and non-violent healthy participants (HC). The main findings were (I) an overall heterogeneous pattern of deviations on the individual level, with a (II) a higher number of extreme negative deviations in SSD-V, SSD-NV, and nonSSD-V than HC, (III) which were most frequent in regions within the collateral transverse sulcus, lingual gyrus, and the cerebellum in SSD-V, a pattern that differed from SSD-NV and nonSSD-V. The study demonstrates the use of normative modeling to map individual brain morphological heterogeneity in forensic psychiatry populations, which has potential to serve as a powerful tool to investigate underlying mechanisms of violent behavior in schizophrenia spectrum disorders and provide clinicians with individual level information that may inform targeted treatment choices.

We report heterogeneous patterns of both extreme and subtle brain deviations associated with violence and schizophrenia spectrum disorders, which is in line with previous studies applying normative models to study brain heterogeneity at the individual level in schizophrenia [23], first episode psychosis [24], and conduct disorder [25]. SSD-V showed more extreme negative deviations in the area of regions within the collateral sulci bilaterally and the adjacent left lingual gyrus [43]. These regions are all located in the basal temporal-occipital lobes, show heritable and sex-specific morphology [50], and are involved in visual processing, including visual perception, object recognition, memory, and integration [51]. A recent meta-analysis showed aberrant task-based functional connectivity in the lingual gyrus associated with violence and psychosis [52] and smaller volume of specifically the left lingual gyrus has been associated with aggression in persons with first episode or high risk for psychosis [53]. Moreover, the SSD-V also showed more extreme deviations in the cerebellar cortex. The cerebellum is important to motor control, cognition, and executive control [54], but has also been linked to aggressive behavior [55, 56]. Taken together, the pattern of deviations in SSD-V point towards regions already known to be associated with violence, aggression and social interaction, but also visual processes which have been linked to delusion formation [57] and social cognitive performance [58]. Indeed, in a subject sample overlapping with the current, we found social cognitive impairments in body emotional perception and theory of mind [33], which associations to underpinning neurobiological mechanisms need to be further explored, but also presents as targets for tailored treatment and training programs at the individual level.

Notably, the SSD-V deviation patterns differed from SSD-NV who showed a higher number of extreme negative deviations in the parieto-occipital area involved in spatial navigation, analysis of visual motor cues and visuomotor limb action control [59], and the suborbital sulci area whose function is less clear, as is its role in schizophrenia psychopathology [60]. While the percentages of patients with extreme deviations were similar to a recent first episode psychosis study applying normative models to cortical thickness [19], the regional patterns differed. This illustrates the inter-individual heterogeneity between modalities (cortical area vs thickness) and study cohorts, and the propensity of the normative models to capture this variance and heterogeneity from a predefined norm [22, 61] without having “failed” to replicate results from earlier studies. Accordingly, the present results are not overlapping with our hypotheses (based on earlier studies), which illustrates that the normative modeling indeed provides an alternative to the traditional case-control group-based studies.

In contrast to both SSD-V and SSD-NV, the nonSSD-V showed more extreme negative deviations in the left paracentral lobule and sulcus and middle frontal sulcus, associated with altered intrinsic brain activity in conduct disorder [62] and emotional dysregulation and inhibitory control [63]. Violence and aggression are indeed heterogeneous constructs comprising different behaviors, psychological profiles, situational characteristics, and sometimes mental disorders [64] and psychopathy traits [65]. Despite the overlapping behavioural characteristics between the nonSSD-V and SSD-V groups, the observed neurobiological heterogeneity and low percentage of overlap at the regional level may reflect effects of psychopathological factors. This low overlap is consistent with findings in schizophrenia spectrum disorders (SSD) more generally, where, as Wolfers et al. reported, an overlap among patients was observed in only a few regions, primarily in frontal, temporal, and cerebellar regions that exceeded 2%, despite robust group differences reported in case control settings [23]. Given the inherent heterogeneity of the underlying factors contributing to violence, it is not surprising that we observe similarly diverse and non-overlapping results in our analysis. Moreover, although SSD-V and nonSSD-V showed similar levels of psychopathy traits, we found no significant associations between psychopathy scores and deviation patterns. There was however a positive trend (significance at the uncorrected level) in the supramarginal gyrus area, which is involved in emotion processing [66], that could be a target for treatment among persons with violence-proneness independent of a comorbid schizophrenia spectrum disorder.

The study has some limitations. The relatively small number of subjects with a history of violence, as well as the male only sample (which reflects the predominance of men in the study group of interest), might limit the generalizability of the current study. However, the normative models are developed to assess exactly the individual brain characteristics in relation to predefined sex-specific charts. Although the Freesurfer Destrieux atlas comprises 148 cortical ROIs, atlas defined cortical parcellations have shown lower representations of true associations and prediction accuracy than vertex-wise analyses [67]. Moreover, the models were not corrected for substance use which could affect brain morphological characteristics, and although persons in the preventive detention facilities and security psychiatry hospital wards should not have access to un-prescribed and illegal substances and alcohol, we cannot exclude potential effects of earlier or non-regulation use. Further, we did not control for cumulative effects of antipsychotic medication which may also affect brain morphology but are also overlapping with clinical groups and their associated psychopathological characteristics. The most severely ill or aggressive persons in the forensic psychiatry wards or prisons were not eligible for participation due to lack of ability to consent or/and safety concerns. Violence was defined as a lifetime binary variable which was narrower than the WHO definition. Despite being narrower, the definition we used is both rigorous and unambiguous, ensuring clear inclusion criteria to groups with a history of violence. Finally, an inherent limitation in all cross-sectional neuroimaging studies is the inability to distinguish between specific disease mechanisms and the effects of early-life influences and developmental trajectories. Cross-sectional normative modeling has shown lower individual level prediction propensity than longitudinal models [68], which should be investigated in future studies of violence and psychosis.

A strength of the current study is that the normative charts are derived from >50 000 individuals based on this Freesurfer standard atlas [20] which ensures that the results are reproducible and easy to compare and facilitates replication studies. The forensic psychiatry patients, prisoners, and regular patients all underwent thorough clinical and socio-demographic characterization, and the prisoners represent almost 20% of all persons serving preventive detention in Norway. The study encompassed a broad age range providing comprehensive coverage across a significant fraction of the lifespan.

In conclusion, this study demonstrates the feasibility of normative modeling to map the heterogeneous individual patterns of brain morphometry deviations associated with violence and psychosis and expands upon the observed patterns of regional group differences identified in traditional case-control studies. While the results warrant replication and further integration with environmental and psychological variables, studies addressing individual brain deviations may contribute to improved understanding of the complex and multidimensional underpinnings of violence in forensic psychiatry and ultimately provide clinicians with individual-level neurobiological information that together with other clinical parameters may contribute to improved treatment and targeted interventions for violence prevention.

## Supplementary information

Supplementary material

## Data Availability

Due to ethical and data security issues related to the sensitive nature of the clinical data, data cannot be shard without specific IRB approval and data use agreements with the relevant institution.
