# Supplementary material for: Individual-level deviations from normative brain morphology in violence, psychosis, and psychopathy
Source: Transl Psychiatry. 2025 Apr 2;15:118. doi: 10.1038/s41398-025-03343-1 (PMC11965457; doi:10.1038/s41398-025-03343-1)
Supplement: Supplementary file 1 — Supplementary material [file 41398_2025_3343_MOESM1_ESM.pdf]

Supplementary material

Supplementary figures

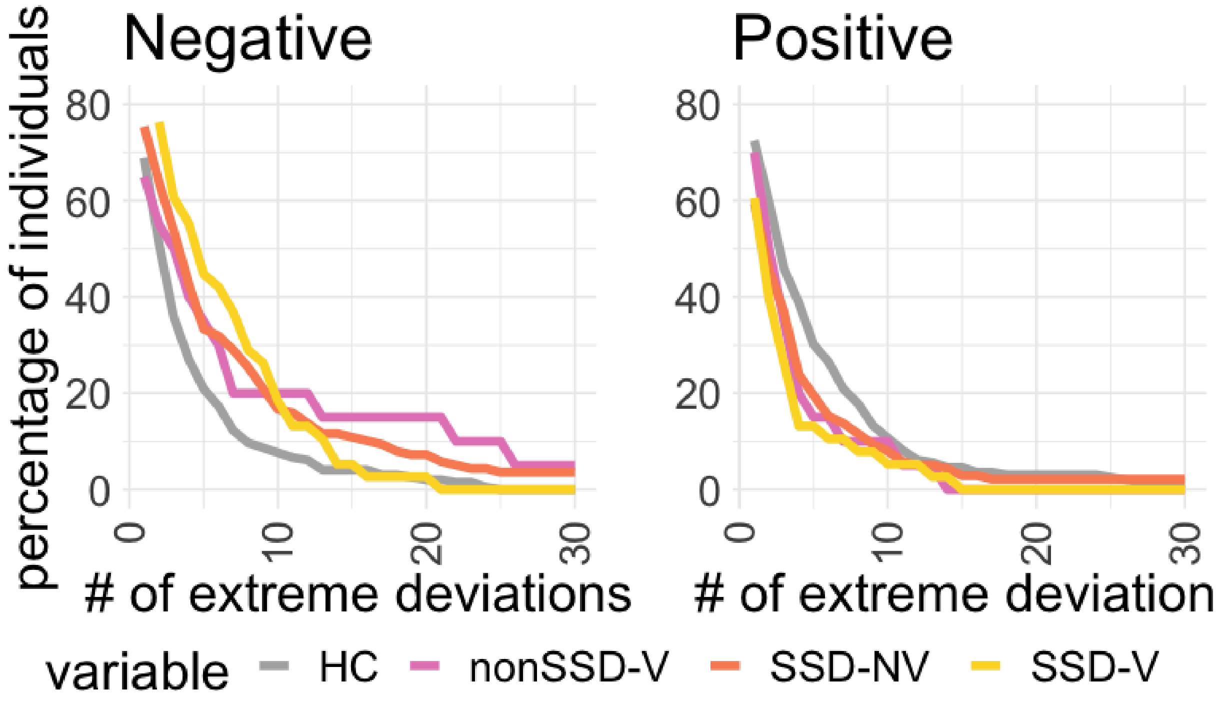

Supplemental figure 1. Association between the percentage of individuals as and the frequency of extreme negative (left) or positive (right) deviations in diagnostic categories. Notably, in the SSD-V group, the prevalence of extreme negative deviations approaches that of HC when the number of outliers reaches 15, whereas in the SSD-NV and nonSSD-V groups, it remains elevated.

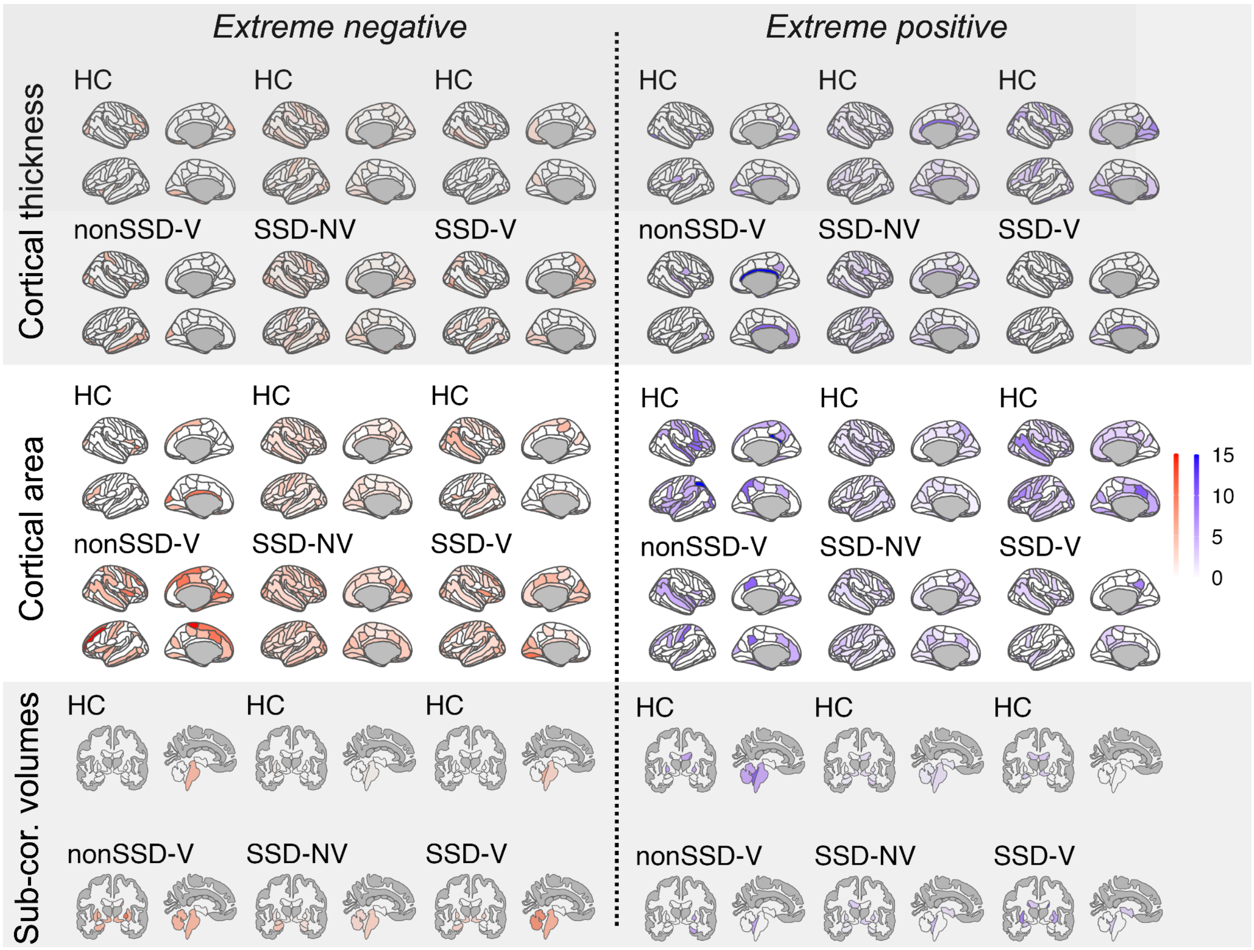

Supplementary figure 2. Extreme negative (left) and positive (right) deviations of cortical thickness (top), area (middle) and subcortical volumes (bottom). Color fill represents percentage of participants from total in the group in given ROI. Diagnostic groups (DX, bottom row within each shaded rectangle) are compared against age matched HC (top row) with ratio 1:1. Extreme deviation is defined as |*Z*|>2.

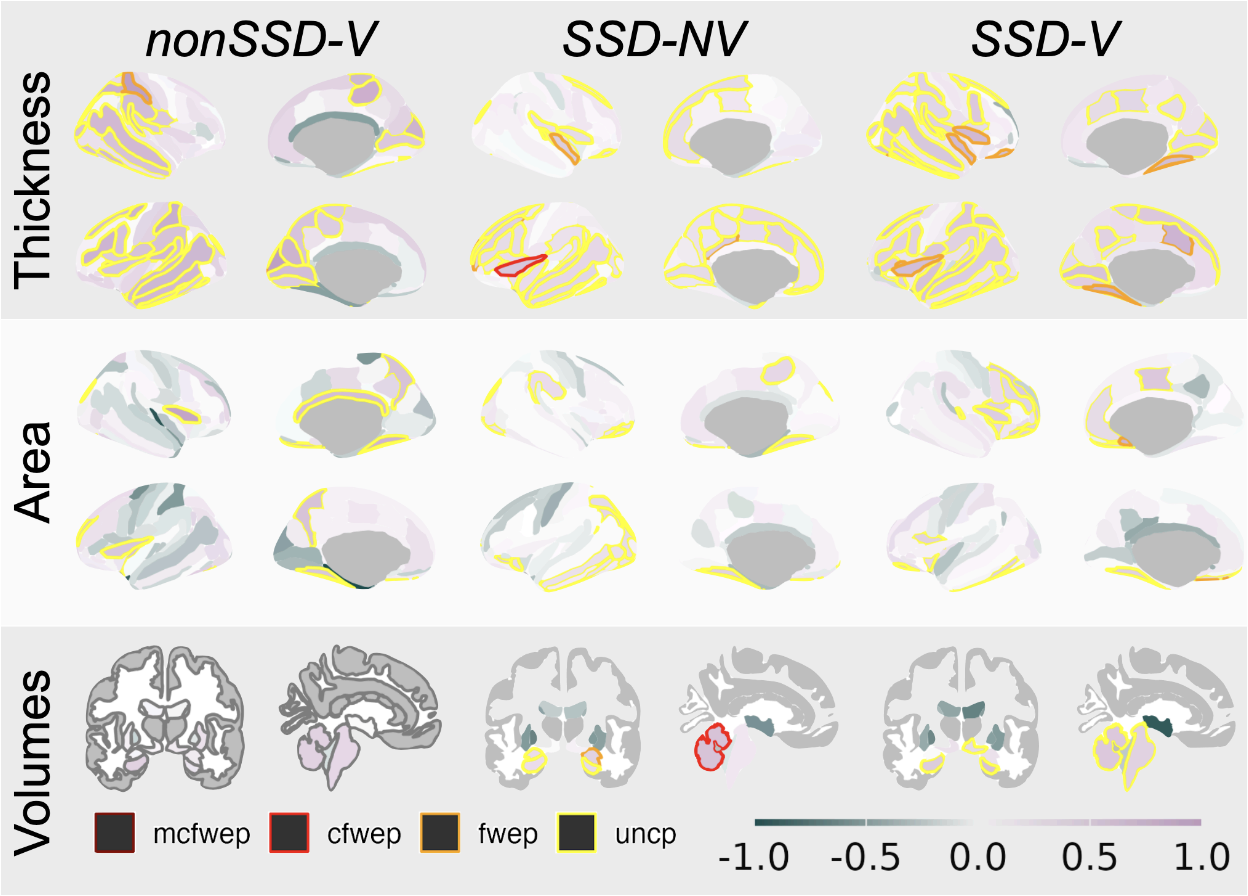

Supplementary figure 3. Pairwise difference in Z-scores between diagnostic groups and age matched to all healthy controls (*n*=196). ROIs with significant results are marked with contour lines: *yellow (uncp)* - uncorrected, *orange (fwep)* - FDR corrected for the number of ROIs, *red (cfwep)* - FDR corrected for the number of ROIs and number of contrasts and *crimson (mcwep)* - p-value FDR corrected for number of ROIs, contrast and modalities. Shades presents Cohen's *d* of group differences on the deviation scores.

Supplementary tables

Supplemental table 1. For each group, we calculated the percentage of extreme negative and positive deviations, defined as $|Z|>2$, for each ROI. The header of each modality indicates the total number of ROIs. Our results showed that all three diagnostic groups exhibited a higher frequency of negative outliers in area and volume, as compared to healthy controls, but not in cortical thickness. Moreover, these diagnostic groups had either fewer or similar instances of extreme positive deviations across all three modalities. *Abbreviations:* DX - diagnosis, SSD-NV - schizophrenia spectrum disorder patients without history of violence, SSD-V - schizophrenia spectrum disorder patients with a history of violence, nonSSD-V - participants with history of violence and no schizophrenia spectrum disorder, HC - healthy controls.

	**Negative**				**Positive**			
**DX**	**Area** *n*=150	**Thick.** *n*=150	**Volume** *n*=32	**Total** *n*=332	**Area** *n*=150	**Thickness** *n*=150	**Volume** *n*=32	**Total** *n*=332
**HC**	1.51	0.95	0.59	1.17	1.56	1.61	1.18	1.55
**nonSSD-V**	3.07	0.93	2.97	2.09	1.20	0.93	0.94	1.05
**SSD-NV**	3.07	1.33	1.52	2.13	1.23	1.30	1.11	1.25
**SSD-V**	2.58	1.18	2.14	1.90	0.68	0.75	2.14	0.86

Supplemental table 2. Pairwise comparisons of frequency of extreme deviations between clinical groups in individuals, significant results are marked in bold. *Abbreviations:* SSD-NV - schizophrenia spectrum disorder patients without history of violence, SSD-V - schizophrenia spectrum disorder patients with a history of violence, nonSSD-V - participants with history of violence and no schizophrenia spectrum disorder, HC - healthy controls.

**Contrast**	$p_{\text{uncorrected}}$	p_{fwe}	**Cohen's *d***
	**Extreme negative deviations**		
HC<NPV	0.0442	0.0850	0.56
HC>NPV	0.9559	0.9985	-0.56
HC<SSD-NV	**0.0035**	**0.0086**	**0.31**
HC>SSD-NV	0.9966	1.0000	-0.31
HC<SSD-V	0.0425	0.0763	0.43
HC>SSD-V	0.9576	0.9989	-0.43
nonSSD-V<SSD-V	0.1952	0.3822	0.20
nonSSD-V>SSD-V	0.8049	0.9618	-0.20
	**Extreme positive deviations**		
HC<NPV	0.8498	0.9840	-0.30
HC>NPV	0.1503	0.2642	0.30
HC<SSD-NV	0.7108	0.9233	-0.07
HC>SSD-NV	0.2893	0.5078	0.07
HC<SSD-V	0.9905	0.9999	-0.58
HC>SSD-V	**0.0096**	**0.0190**	**0.58**
nonSSD-V<SSD-V	0.3240	0.5373	0.11
nonSSD-V>SSD-V	0.6761	0.9110	-0.11

Supplemental table 3. Frequency of individuals per diagnostic category exhibiting 1 to 30 extreme negative deviations, summarized by modalities: cortical area and thickness, subcortical volumes, and the total number across all modalities.

	Cortical Area (*n* = 150)				Cortical Thickness (*n* = 150)				Subcortical volume (*n* = 32)				Total (*n* = 332)			
**Deviations**	**HC**	**nonSSD-V**	**SSD-NV**	**SSD-V**	**HC**	**nonSSD-V**	**SSD-NV**	**SSD-V**	**HC**	**nonSSD-V**	**SSD-NV**	**SSD-V**	**HC**	**nonSSD-V**	**SSD-NV**	**SSD-V**
**1**	43.9	40.0	54.3	68.4	29.6	35.0	37.7	39.5	2.0	10.0	10.1	15.8	68.9	65.0	75.4	89.5
**2**	26.5	35.0	45.7	47.4	15.8	20.0	25.4	23.7	0.0	5.0	6.5	10.5	51.0	55.0	63.8	76.3
**3**	17.3	30.0	34.1	42.1	9.7	10.0	15.9	15.8	0.0	5.0	2.9	7.9	35.7	50.0	53.6	60.5
**4**	14.3	25.0	25.4	42.1	8.2	10.0	7.2	13.2	0.0	5.0	0.7	2.6	27.0	40.0	42.8	55.3
**5**	12.8	15.0	20.3	28.9	5.6	5.0	4.3	10.5	0.0	5.0	0.7	2.6	20.9	35.0	33.3	44.7
**6**	8.2	15.0	17.4	21.1	4.6	5.0	3.6	5.3	0.0	5.0	0.7	2.6	17.3	30.0	31.9	42.1
**7**	7.1	15.0	15.9	21.1	3.1	0.0	3.6	5.3	0.0	5.0	0.7	0.0	12.2	20.0	29.0	36.8
**8**	6.1	15.0	15.2	13.2	3.1	0.0	3.6	2.6	0.0	5.0	0.7	0.0	9.7	20.0	25.4	28.9
**9**	3.6	15.0	13.0	10.5	2.6	0.0	3.6	0.0	0.0	5.0	0.7	0.0	8.7	20.0	21.0	26.3
**10**	3.6	15.0	11.6	2.6	2.0	0.0	3.6	0.0	0.0	5.0	0.7	0.0	7.7	20.0	16.7	18.4
**11**	2.6	15.0	10.1	2.6	1.5	0.0	2.9	0.0	0.0	5.0	0.7	0.0	6.6	20.0	15.9	13.2
**12**	2.0	15.0	9.4	2.6	1.5	0.0	2.9	0.0	0.0	5.0	0.7	0.0	6.1	20.0	13.8	13.2
**13**	1.5	10.0	6.5	2.6	1.5	0.0	2.9	0.0	0.0	5.0	0.7	0.0	4.1	15.0	11.6	10.5
**14**	1.5	10.0	6.5	2.6	1.0	0.0	2.9	0.0	0.0	5.0	0.0	0.0	4.1	15.0	11.6	5.3
**15**	1.0	10.0	6.5	0.0	1.0	0.0	2.9	0.0	0.0	0.0	0.0	0.0	4.1	15.0	10.9	5.3

**16**	1.0	10.0	5.8	0.0	1.0	0.0	2.2	0.0	0.0	0.0	0.0	0.0	4.1	15.0	10.1	2.6
**17**	1.0	10.0	5.8	0.0	0.5	0.0	2.2	0.0	0.0	0.0	0.0	0.0	3.1	15.0	9.4	2.6
**18**	1.0	10.0	5.8	0.0	0.0	0.0	2.2	0.0	0.0	0.0	0.0	0.0	3.1	15.0	8.0	2.6
**19**	1.0	10.0	5.8	0.0	0.0	0.0	2.2	0.0	0.0	0.0	0.0	0.0	2.6	15.0	7.2	2.6
**20**	0.5	10.0	3.6	0.0	0.0	0.0	2.2	0.0	0.0	0.0	0.0	0.0	2.0	15.0	7.2	2.6
**21**	0.5	10.0	2.9	0.0	0.0	0.0	2.2	0.0	0.0	0.0	0.0	0.0	2.0	15.0	5.8	0.0
**22**	0.5	10.0	2.2	0.0	0.0	0.0	0.7	0.0	0.0	0.0	0.0	0.0	1.5	10.0	5.1	0.0
**23**	0.5	10.0	2.2	0.0	0.0	0.0	0.7	0.0	0.0	0.0	0.0	0.0	1.5	10.0	4.3	0.0
**24**	0.0	10.0	2.2	0.0	0.0	0.0	0.7	0.0	0.0	0.0	0.0	0.0	0.5	10.0	4.3	0.0
**25**	0.0	10.0	2.2	0.0	0.0	0.0	0.7	0.0	0.0	0.0	0.0	0.0	0.0	10.0	3.6	0.0
**26**	0.0	5.0	2.2	0.0	0.0	0.0	0.7	0.0	0.0	0.0	0.0	0.0	0.0	5.0	3.6	0.0
**27**	0.0	5.0	1.4	0.0	0.0	0.0	0.7	0.0	0.0	0.0	0.0	0.0	0.0	5.0	3.6	0.0
**28**	0.0	5.0	1.4	0.0	0.0	0.0	0.0	0.0	0.0	0.0	0.0	0.0	0.0	5.0	3.6	0.0
**29**	0.0	5.0	1.4	0.0	0.0	0.0	0.0	0.0	0.0	0.0	0.0	0.0	0.0	5.0	3.6	0.0
**30**	0.0	0.0	1.4	0.0	0.0	0.0	0.0	0.0	0.0	0.0	0.0	0.0	0.0	5.0	3.6	0.0

Supplemental table 4. Frequency of individuals per diagnostic category exhibiting 1 to 30 extreme positive deviations, summarized by modalities: cortical area and thickness, subcortical volumes, and the total number across all modalities.

	**Cortical Area (*n* = 150)**				**Cortical Thickness (*n* = 150)**				**Subcortical volume (*n* = 32)**				**Total (*n* = 332)**			
**Deviations**	**HC**	**nonSSD-V**	**SSD-NV**	**SSD-V**	**HC**	**nonSSD-V**	**SSD-NV**	**SSD-V**	**HC**	**nonSSD-V**	**SSD-NV**	**SSD-V**	**HC**	**nonSSD-V**	**SSD-NV**	**SSD-V**
**1**	35.2	45.0	29.0	18.4	46.4	30.0	29.7	31.6	8.2	5.0	8.0	18.4	72.4	70.0	59.4	60.5
**2**	24.5	20.0	18.1	10.5	32.1	15.0	20.3	15.8	5.1	0.0	2.9	10.5	59.7	50.0	45.7	39.5
**3**	15.8	10.0	11.6	10.5	21.4	5.0	15.2	5.3	2.0	0.0	2.2	5.3	45.9	35.0	37.0	26.3
**4**	13.3	5.0	7.2	7.9	17.3	5.0	12.3	0.0	1.0	0.0	1.4	5.3	38.8	20.0	23.9	13.2
**5**	10.7	5.0	6.5	5.3	13.3	5.0	9.4	0.0	1.0	0.0	0.7	5.3	30.1	15.0	19.6	13.2
**6**	6.6	5.0	4.3	5.3	10.7	5.0	5.8	0.0	0.5	0.0	0.7	0.0	26.5	15.0	15.2	10.5
**7**	5.1	5.0	4.3	5.3	9.2	5.0	5.1	0.0	0.5	0.0	0.0	0.0	20.9	10.0	13.8	10.5
**8**	4.1	5.0	4.3	2.6	6.1	5.0	4.3	0.0	0.5	0.0	0.0	0.0	17.9	10.0	11.6	7.9
**9**	3.6	5.0	3.6	2.6	4.1	5.0	2.9	0.0	0.0	0.0	0.0	0.0	13.3	10.0	9.4	7.9
**10**	3.6	5.0	2.9	0.0	2.6	0.0	2.9	0.0	0.0	0.0	0.0	0.0	10.7	10.0	8.0	5.3
**11**	3.1	5.0	2.2	0.0	2.0	0.0	2.2	0.0	0.0	0.0	0.0	0.0	8.2	5.0	5.8	5.3
**12**	3.1	0.0	2.2	0.0	1.5	0.0	2.2	0.0	0.0	0.0	0.0	0.0	6.1	5.0	5.1	5.3
**13**	3.1	0.0	1.4	0.0	1.5	0.0	2.2	0.0	0.0	0.0	0.0	0.0	5.6	5.0	5.1	2.6
**14**	3.1	0.0	0.7	0.0	1.5	0.0	2.2	0.0	0.0	0.0	0.0	0.0	4.6	0.0	4.3	2.6
**15**	2.6	0.0	0.7	0.0	0.5	0.0	1.4	0.0	0.0	0.0	0.0	0.0	4.6	0.0	2.9	0.0

**16**	2.6	0.0	0.7	0.0	0.5	0.0	1.4	0.0	0.0	0.0	0.0	0.0	3.6	0.0	2.9	0.0
**17**	2.6	0.0	0.7	0.0	0.5	0.0	1.4	0.0	0.0	0.0	0.0	0.0	3.6	0.0	2.2	0.0
**18**	2.6	0.0	0.7	0.0	0.5	0.0	1.4	0.0	0.0	0.0	0.0	0.0	3.1	0.0	2.2	0.0
**19**	2.6	0.0	0.7	0.0	0.5	0.0	1.4	0.0	0.0	0.0	0.0	0.0	3.1	0.0	2.2	0.0
**20**	2.6	0.0	0.7	0.0	0.5	0.0	1.4	0.0	0.0	0.0	0.0	0.0	3.1	0.0	2.2	0.0
**21**	2.0	0.0	0.7	0.0	0.5	0.0	1.4	0.0	0.0	0.0	0.0	0.0	3.1	0.0	2.2	0.0
**22**	2.0	0.0	0.7	0.0	0.5	0.0	1.4	0.0	0.0	0.0	0.0	0.0	3.1	0.0	2.2	0.0
**23**	1.0	0.0	0.7	0.0	0.5	0.0	1.4	0.0	0.0	0.0	0.0	0.0	3.1	0.0	2.2	0.0
**24**	1.0	0.0	0.7	0.0	0.5	0.0	1.4	0.0	0.0	0.0	0.0	0.0	3.1	0.0	2.2	0.0
**25**	1.0	0.0	0.7	0.0	0.5	0.0	1.4	0.0	0.0	0.0	0.0	0.0	2.6	0.0	2.2	0.0
**26**	1.0	0.0	0.7	0.0	0.0	0.0	1.4	0.0	0.0	0.0	0.0	0.0	2.0	0.0	2.2	0.0
**27**	1.0	0.0	0.7	0.0	0.0	0.0	1.4	0.0	0.0	0.0	0.0	0.0	1.5	0.0	2.2	0.0
**28**	1.0	0.0	0.7	0.0	0.0	0.0	1.4	0.0	0.0	0.0	0.0	0.0	1.5	0.0	2.2	0.0
**29**	1.0	0.0	0.7	0.0	0.0	0.0	1.4	0.0	0.0	0.0	0.0	0.0	1.5	0.0	2.2	0.0
**30**	1.0	0.0	0.7	0.0	0.0	0.0	1.4	0.0	0.0	0.0	0.0	0.0	1.5	0.0	2.2	0.0

Supplemental table 5. Regions with the highest percentage of individuals with extreme negative deviations, stratified by diagnostic groups. The table is arranged in descending order based on percentages in the SSD-V column. *Abbreviations:* SSD-NV - schizophrenia spectrum disorder patients without history of violence, SSD-V - participants with history of violence and schizophrenia spectrum disorder, nonSSD-V - participants with history of violence, but without schizophrenia spectrum disorder, HC - healthy controls.

**Hemi**	**Region**	**Modality**	**SSD-V**	**SSD-NV**	**nonSSD-V**	**HC**
Left	S_collat_transv_ant	Area	13.2	2.9	0.0	2.0
Right	S_collat_transv_post	Area	10.5	3.6	5.0	4.6
Left	G_oc.temp_med.Lingual	Area	10.5	3.6	5.0	1.5
Right	Cerebellum.Cortex	Volume	10.5	2.9	5.0	1.0
Right	S_collat_transv_ant	Area	10.5	2.9	0.0	2.0
Left	Cerebellum.White.Matter	Volume	10.5	2.2	5.0	0.0
Right	G_front_middle	Area	7.9	6.5	10.0	2.0
Left	G_cuneus	Area	7.9	5.1	0.0	2.6
Left	S_orbital_lateral	Area	7.9	4.3	5.0	2.6
Right	S_circular_insula_sup	Area	7.9	3.6	10.0	1.0
Left	S_oc.temp_lat	Area	7.9	3.6	0.0	0.5
Right	Pole_occipital	Area	7.9	2.9	5.0	0.5
Right	S_orbital.H_Shaped	Area	7.9	2.9	0.0	1.5
Right	G_Ins_lg_and_S_cent_ins	Area	7.9	2.2	10.0	0.0
Right	Cerebellum.White.Matter	Volume	7.9	2.2	5.0	0.0

Supplemental table 6. Regions with the highest percentage of individuals with extreme negative deviations, focusing exclusively on diagnostic groups. The table is arranged in descending order based on percentages in the SSD-NV column. *Abbreviations:* SSD-NV - schizophrenia spectrum disorder patients without history of violence, SSD-V - participants with history of violence and schizophrenia spectrum disorder, nonSSD-V - participants with history of violence, but without schizophrenia spectrum disorder, HC - healthy controls.

**Hemi**	**Region**	**Modality**	**SSD-NV**	**SSD-V**	**nonSSD-V**	**HC**
Left	Vessel	Volume	9.4	2.6	5.0	4.6
Right	S_parieto_occipital	Area	7.2	5.3	5.0	1.0
Right	Lat_Fis.ant.Vertical	Area	7.2	2.6	10.0	2.0
Right	S_suborbital	Area	7.2	2.6	0.0	6.6
Right	Lat_Fis.ant.Horizont	Area	7.2	2.6	0.0	4.1
Right	S_oc_sup_and_transversal	Area	7.2	0.0	10.0	3.1
Right	G_front_middle	Area	6.5	7.9	10.0	2.0
Right	G_occipital_middle	Area	6.5	5.3	5.0	1.0
Left	G_occipital_middle	Area	6.5	5.3	0.0	1.0
Left	S_oc_middle_and_Lunatus	Area	6.5	2.6	0.0	2.0
Right	G_cuneus	Area	5.8	5.3	0.0	1.0
Right	S_oc_middle_and_Lunatus	Area	5.8	5.3	0.0	1.5
Left	G_subcallosal	Thickness	5.8	2.6	0.0	0.5
Right	G_temporal_inf	Area	5.8	2.6	0.0	0.5
Left	S_temporal_inf	Area	5.8	0.0	5.0	3.6

Supplemental table 7. Regions with the highest percentage of individuals with extreme negative deviations, stratified by diagnostic groups. The table is arranged in descending order based on percentages in the nonSSD-V column. *Abbreviations:* SSD-NV - schizophrenia spectrum disorder patients without history of violence, SSD-V - participants with history of violence and schizophrenia spectrum disorder, nonSSD-V - participants with history of violence, but without schizophrenia spectrum disorder, HC - healthy controls.

Hemi	Region	Modality	nonSSD-V	SSD-V	SSD-NV	HC
Left	G_and_S_paracentral	Area	20.0	0.0	2.2	0.5
Left	G_front_middle	Area	15.0	5.3	3.6	1.0
Right	Lat_Fis.ant.Vertical	Area	10.0	2.6	7.2	2.0
Right	S_oc_sup_and_transversal	Area	10.0	0.0	7.2	3.1
Right	G_front_middle	Area	10.0	7.9	6.5	2.0
Right	G_oc.temp_lat.fusifor	Area	10.0	2.6	5.1	3.1
Left	G_oc.temp_lat.fusifor	Thickness	10.0	0.0	4.3	1.5
Right	S_circular_insula_sup	Area	10.0	7.9	3.6	1.0
Left	G_and_S_cingul.Mid.Ant	Area	10.0	2.6	3.6	1.0
Right	G_occipital_sup	Area	10.0	0.0	3.6	2.6
Right	G_and_S_cingul.Mid.Ant	Area	10.0	5.3	2.9	0.0
Left	G_occipital_sup	Thickness	10.0	2.6	2.9	1.5
Left	S_front_sup	Area	10.0	2.6	2.9	1.0
Right	G_front_sup	Area	10.0	0.0	2.9	2.0
Left	G_Ins_lg_and_S_cent_ins	Area	10.0	7.9	2.2	0.0

Supplemental table 8. The comparison of deviations between diagnostic groups and healthy participants, matched 1:1 by age yielded four distinct p-values, each accounting for varying levels of multiple comparison corrections: *'unc'* denotes the uncorrected p-value; *'fwe'* represents the p-value corrected for the false discovery rate (FDR) considering the number of regions of interest (ROIs); *'cfwe'* indicates the FDR-corrected p-value taking into account both the number of ROIs and the number of contrasts; and *'mcfwe'* stands for the FDR-corrected p-value adjusted for the number of ROIs, the number of contrasts, and the number of imaging modalities. *Abbreviations:* SSD-NV - schizophrenia spectrum disorder patients without history of violence, SSD-V - participants with history of violence and schizophrenia spectrum disorder, nonSSD-V - participants with history of violence, but without schizophrenia spectrum disorder, HC - healthy controls.

Hemi	ROI	Modality	Contrast	P_{unc}	P_{fwe}	P_{cfwe}	P_{mcfwe}	t-value	Cohen's d
Right	G_subcallosal	area	SSD-V < HC	1.00E-04	8.00E-04	1.03E-02	2.05E-02	4.53	1.17
Left	Inf_Lat_Vent	volume	SSD-NV > HC	1.00E-04	1.10E-03	8.50E-03	6.78E-02	4.22	0.61
Left	S_oc_temp_med_and_Lingual	thickness	SSD-V < HC	1.00E-04	2.20E-03	2.20E-02	5.22E-02	4.30	0.99
Left	Pallidum	volume	SSD-NV > HC	3.00E-04	2.60E-03	2.45E-02	1.76E-01	3.96	0.57
Right	Cerebellum_Cortex	volume	SSD-NV < HC	2.00E-04	4.30E-03	5.84E-02	3.94E-01	3.69	0.53
Left	S_oc_temp_med_and_Lingual	thickness	nonSSD-V > SSD-V	1.00E-04	5.40E-03	6.06E-02	1.48E-01	4.01	1.22
Left	Cerebellum_White_Matter	volume	SSD-V < HC	2.00E-04	6.10E-03	7.82E-02	4.85E-01	3.60	0.93
Left	Cerebellum_Cortex	volume	SSD-NV < HC	2.00E-04	6.40E-03	8.32E-02	5.08E-01	3.58	0.51
Left	G_and_S_cingul_Mid_Ant	thickness	SSD-NV < HC	3.00E-04	8.60E-03	8.87E-02	2.19E-01	3.89	0.49
Right	S_circular_insula_inf	thickness	SSD-V < HC	2.00E-04	1.08E-02	1.03E-01	2.50E-01	3.85	0.89

Right	Cerebellum_White_Matter	volume	SSD-V < HC	8.00E-04	1.12E-02	1.46E-01	7.14E-01	3.40	0.88
Left	Putamen	volume	SSD-NV > HC	7.00E-04	1.31E-02	1.77E-01	7.89E-01	3.33	0.48
Left	choroid_plexus	volume	SSD-V > HC	7.00E-04	1.51E-02	2.07E-01	8.44E-01	3.27	0.84
Right	Inf_Lat_Vent	volume	SSD-V > HC	8.00E-04	1.81E-02	2.41E-01	8.92E-01	3.21	0.83
Bi	X3rd_Ventricle	volume	SSD-NV > HC	1.00E-03	2.04E-02	2.44E-01	8.97E-01	3.21	0.46
Left	S_front_sup	thickness	SSD-NV < HC	1.00E-04	2.11E-02	2.03E-01	4.45E-01	3.63	0.45
Left	S_calcarine	thickness	SSD-V < HC	4.00E-04	2.37E-02	2.33E-01	4.99E-01	3.59	0.83
Left	S_circular_insula_sup	thickness	SSD-NV < HC	2.00E-04	3.19E-02	2.82E-01	5.79E-01	3.52	0.44
Right	Putamen	volume	SSD-NV > HC	1.10E-03	3.22E-02	3.44E-01	9.68E-01	3.07	0.44
Left	S_collat_transv_ant	area	nonSSD-V > SSD-V	3.00E-04	3.55E-02	3.84E-01	6.02E-01	3.50	1.07
Left	Cerebellum_White_Matter	volume	SSD-NV < HC	1.70E-03	3.86E-02	4.17E-01	9.89E-01	2.97	0.43
Right	Lateral_Ventricle	volume	SSD-V > HC	2.00E-03	3.94E-02	4.17E-01	9.89E-01	2.97	0.77
Bi	X3rd_Ventricle	volume	SSD-V > HC	2.00E-03	3.97E-02	4.19E-01	9.89E-01	2.97	0.77
Right	Pallidum	volume	SSD-NV > HC	1.70E-03	4.13E-02	4.12E-01	9.88E-01	2.98	0.43
Left	S_circular_insula_sup	thickness	SSD-V < HC	6.00E-04	4.18E-02	3.75E-01	7.03E-01	3.41	0.79
Left	MeanThickness	thickness	SSD-V < HC	6.00E-04	4.44E-02	3.94E-01	7.27E-01	3.39	0.78
Left	G_cingul_Post_dorsal	thickness	SSD-NV < HC	3.00E-04	4.54E-02	3.85E-01	7.13E-01	3.40	0.42
Left	S_collat_transv_ant	area	SSD-V < HC	3.00E-04	4.77E-02	4.78E-01	7.07E-01	3.40	0.88

Right	choroid_plexus	volume	SSD-NV < SSD-V	2.00E-03	4.86E-02	4.50E-01	9.93E-01	2.94	0.67
Left	G_front_middle	thickness	SSD-NV < HC	1.00E-03	4.93E-02	4.11E-01	7.48E-01	3.37	0.42

Supplemental table 9. The comparison of deviations between diagnostic groups and all healthy participants (n=196) with age of participant included as a covariate, yielded four distinct p-values, each accounting for varying levels of multiple comparison corrections: *'unc'* denotes the uncorrected p-value; *'fwe'* represents the p-value corrected for the false discovery rate (FDR) considering the number of regions of interest (ROIs); *'cfwe'* indicates the FDR-corrected p-value taking into account both the number of ROIs and the number of contrasts; and *'mcfwe'* stands for the FDR-corrected p-value adjusted for the number of ROIs, the number of contrasts, and the number of imaging modalities. *Abbreviations:* SSD-NV - schizophrenia spectrum disorder patients without history of violence, SSD-V - participants with history of violence and schizophrenia spectrum disorder, nonSSD-V - participants with history of violence, but without schizophrenia spectrum disorder, HC - healthy controls.

**Hemi**	**ROI**	**Modality**	**Contrast**	P_{unc}	P_{fwe}	P_{cfwe}	P_{mcfwe}	**t-value**	**Cohen's d**
Bi	X3rd_Ventricle	volume	SSD-V > HC	1.00E-04	8.00E-04	1.19E-02	8.82E-02	4.14	0.91
Left	Inf_Lat_Vent	volume	SSD-V > HC	1.00E-04	1.00E-03	1.70E-02	1.24E-01	4.04	0.89
Left	Pallidum	volume	SSD-NV > HC	3.00E-04	1.10E-03	1.37E-02	9.74E-02	4.11	0.65
Left	Inf_Lat_Vent	volume	SSD-NV > HC	1.00E-04	1.20E-03	1.49E-02	1.08E-01	4.08	0.65
Left	Cerebellum_Cortex	volume	SSD-NV < HC	1.00E-04	1.20E-03	1.24E-02	9.07E-02	4.13	0.66
Right	Cerebellum_Cortex	volume	SSD-NV < HC	1.00E-04	1.30E-03	1.37E-02	9.75E-02	4.11	0.65
Left	S_circular_insula_sup	thickness	SSD-NV < HC	1.00E-04	2.70E-03	3.10E-02	8.25E-02	4.15	0.59
Right	Inf_Lat_Vent	volume	SSD-V > HC	1.00E-04	4.20E-03	4.62E-02	3.23E-01	3.74	0.82
Left	S_oc_temp_med_and_Lingual	thickness	SSD-V < HC	2.00E-04	5.30E-03	5.30E-02	1.38E-01	4.01	0.80
Left	S_oc_temp_med_and_Lingual	thickness	nonSSD-V > SSD-V	1.00E-04	5.50E-03	5.32E-02	1.39E-01	4.01	1.21

Bi	X3rd_Ventricle	volume	SSD-NV > HC	5.00E-04	6.20E-03	8.15E-02	4.95E-01	3.57	0.57
Right	S_postcentral	thickness	nonSSD-V < HC	1.00E-04	6.70E-03	7.67E-02	1.94E-01	3.91	1.17
Left	G_and_S_cingul_Mid_Ant	thickness	SSD-V < HC	3.00E-04	7.60E-03	8.41E-02	2.10E-01	3.88	0.78
Left	Cerebellum_White_Matter	volume	SSD-NV < HC	3.00E-04	1.13E-02	1.52E-01	7.36E-01	3.35	0.53
Left	Putamen	volume	SSD-NV > HC	8.00E-04	1.16E-02	1.48E-01	7.27E-01	3.36	0.53
Right	S_circular_insula_inf	thickness	SSD-NV < HC	1.00E-04	1.20E-02	1.33E-01	3.12E-01	3.75	0.53
Left	choroid_plexus	volume	SSD-V > HC	6.00E-04	1.27E-02	1.38E-01	7.06E-01	3.39	0.75
Right	Pallidum	volume	SSD-NV > HC	1.00E-03	1.55E-02	1.81E-01	8.03E-01	3.29	0.52
Right	S_circular_insula_inf	thickness	SSD-V < HC	4.00E-04	1.57E-02	1.73E-01	3.95E-01	3.66	0.73
Right	choroid_plexus	volume	SSD-V > HC	7.00E-04	1.63E-02	1.87E-01	8.14E-01	3.28	0.72
Right	G_oc_temp_lat_fusifor	thickness	SSD-V < HC	2.00E-04	1.68E-02	1.83E-01	4.17E-01	3.64	0.73
Left	S_orbital_med_olfact	area	SSD-V < HC	3.00E-04	1.78E-02	1.87E-01	3.28E-01	3.73	0.82
Right	S_circular_insula_sup	thickness	SSD-V < HC	1.00E-04	2.06E-02	2.18E-01	4.78E-01	3.59	0.72
Left	G_cingul_Post_dorsal	thickness	SSD-NV < HC	4.00E-04	2.25E-02	2.08E-01	4.61E-01	3.60	0.51
Right	Putamen	volume	SSD-NV > HC	1.10E-03	2.77E-02	3.06E-01	9.42E-01	3.08	0.49
Left	Caudate	volume	SSD-NV > HC	1.50E-03	3.10E-02	3.28E-01	9.55E-01	3.05	0.48
Left	Lat_Fis_ant_Vertical	thickness	SSD-V < HC	4.00E-04	3.45E-02	3.25E-01	6.38E-01	3.44	0.69

Left	G_oc_temp_med_Parahip	area	nonSSD-V > HC	6.00E-04	3.51E-02	3.54E-01	5.62E-01	3.51	1.06
Right	G_subcallosal	area	SSD-V < HC	5.00E-04	3.59E-02	3.49E-01	5.57E-01	3.52	0.78
Left	S_circular_insula_sup	thickness	SSD-V < HC	5.00E-04	3.61E-02	3.37E-01	6.55E-01	3.43	0.69
Left	MeanThickness	thickness	SSD-V < HC	6.00E-04	3.84E-02	3.57E-01	6.80E-01	3.41	0.68
Right	Lateral_Ventricle	volume	SSD-V > HC	1.50E-03	3.90E-02	3.60E-01	9.69E-01	3.02	0.66
Left	G_cuneus	thickness	nonSSD-V < HC	2.00E-04	4.16E-02	3.71E-01	7.00E-01	3.39	1.01
Right	S_oc_temp_med_and_Lingual	thickness	SSD-V < HC	5.00E-04	4.32E-02	3.87E-01	7.16E-01	3.37	0.68
Right	choroid_plexus	volume	SSD-NV < SSD-V	2.10E-03	4.36E-02	3.92E-01	9.80E-01	2.98	0.67
Left	S_collat_transv_ant	area	nonSSD-V > SSD-V	3.00E-04	4.51E-02	4.36E-01	6.66E-01	3.42	1.04
Left	G_front_middle	thickness	SSD-NV < HC	8.00E-04	4.60E-02	3.85E-01	7.14E-01	3.38	0.48
Right	Amygdala	volume	SSD-NV < HC	1.70E-03	4.77E-02	4.41E-01	9.91E-01	2.92	0.46
Right	S_orbital_H_Shaped	thickness	SSD-V < HC	4.00E-04	4.87E-02	4.20E-01	7.55E-01	3.34	0.67
Right	Inf_Lat_Vent	volume	SSD-NV > HC	2.00E-03	4.93E-02	4.55E-01	9.94E-01	2.90	0.46

Supplemental table 10. Associations between PCL-R scores and deviations values. The top three associations for each modality (area, thickness and volume), as determined by Cohen's d are presented.

**Feature**	**Hemisphere**	**Modality**	**Direction**	**Cohen's d**	**T value**	p_{unc}	p_{fwe}	p_{cfwe}	p_{mcfwe}
S_subparietal	right	area	Inc	1.836	2.9489	0.0041	0.3095	0.6922	0.8916
G_and_S_paracentral	right	area	Dec	1.8012	2.8930	0.0054	0.3602	0.7325	0.9136
G_cingul.Post.dorsal	right	area	Inc	1.7743	2.8498	0.0051	0.3663	0.7633	0.9302
Lateral.Ventricle	right	volume	Dec	1.4417	2.3155	0.0158	0.2811	0.6065	0.9988
VentralDC	right	volume	Inc	1.3595	2.1835	0.0216	0.3388	0.7019	0.9997
Cerebellum.Cortex	left	volume	Inc	1.2099	1.9433	0.0349	0.4797	0.8475	1
G_pariet_inf.Supramar	right	thickness	Inc	1.7759	2.7588	0.0056	0.3748	0.7599	0.9567
G_and_S_subcentral	right	thickness	Inc	1.6644	2.5857	0.0076	0.4705	0.8689	0.9861
G_temp_sup.G_T_transv	right	thickness	Dec	1.5715	2.4413	0.0103	0.5452	0.9360	0.9953
